# Supplementary figures and images for: Multi-Omics Characterization of a Human Stem Cell-Based Model of Cardiac Hypertrophy
Source: Life (Basel). 2022 Feb 16;12(2):293. doi: 10.3390/life12020293 (PMC8875317; doi:10.3390/life12020293)

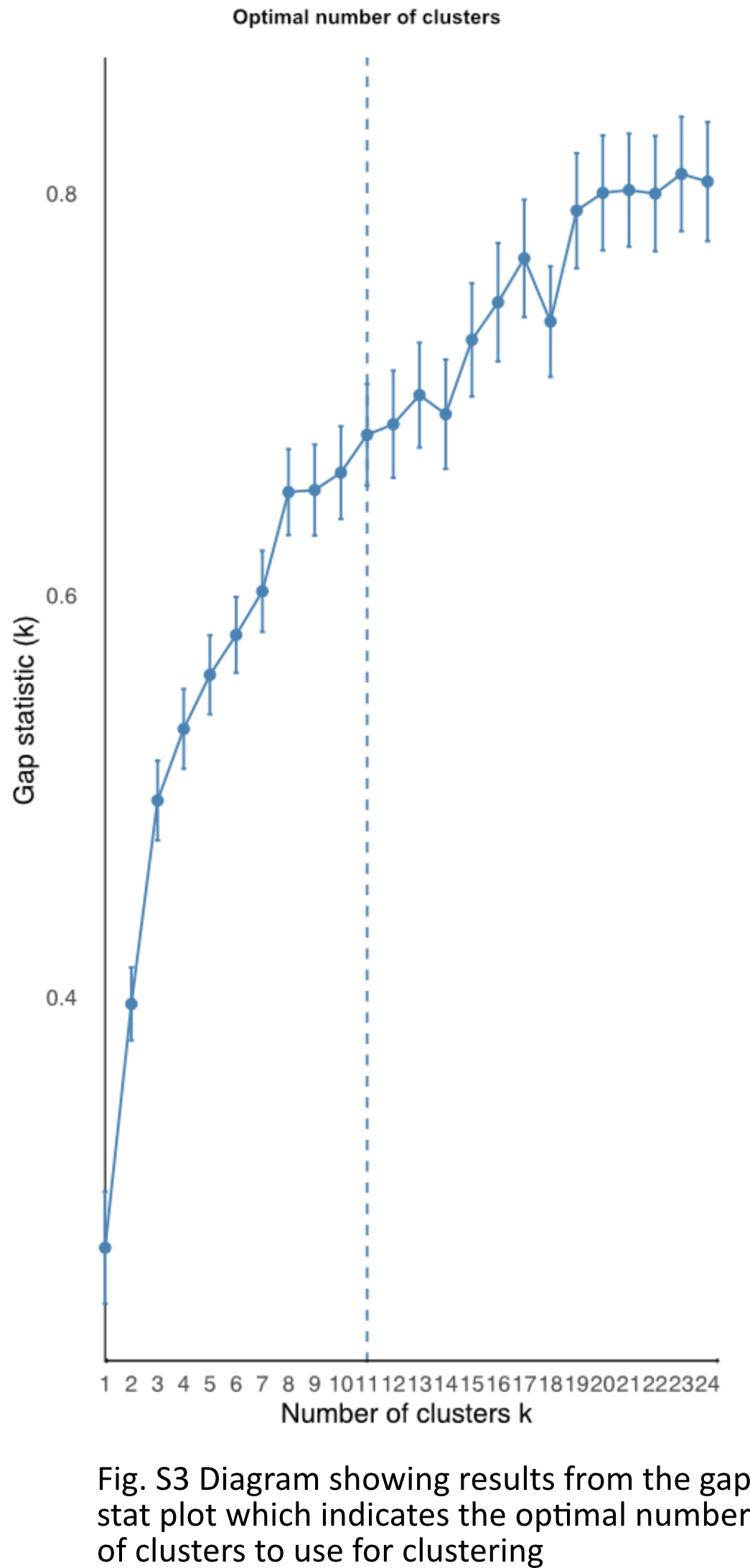

Supplement: Supplementary file 1 [file life-12-00293-s001.zip › life-1577086-supplementary/Figure S1.jpg]

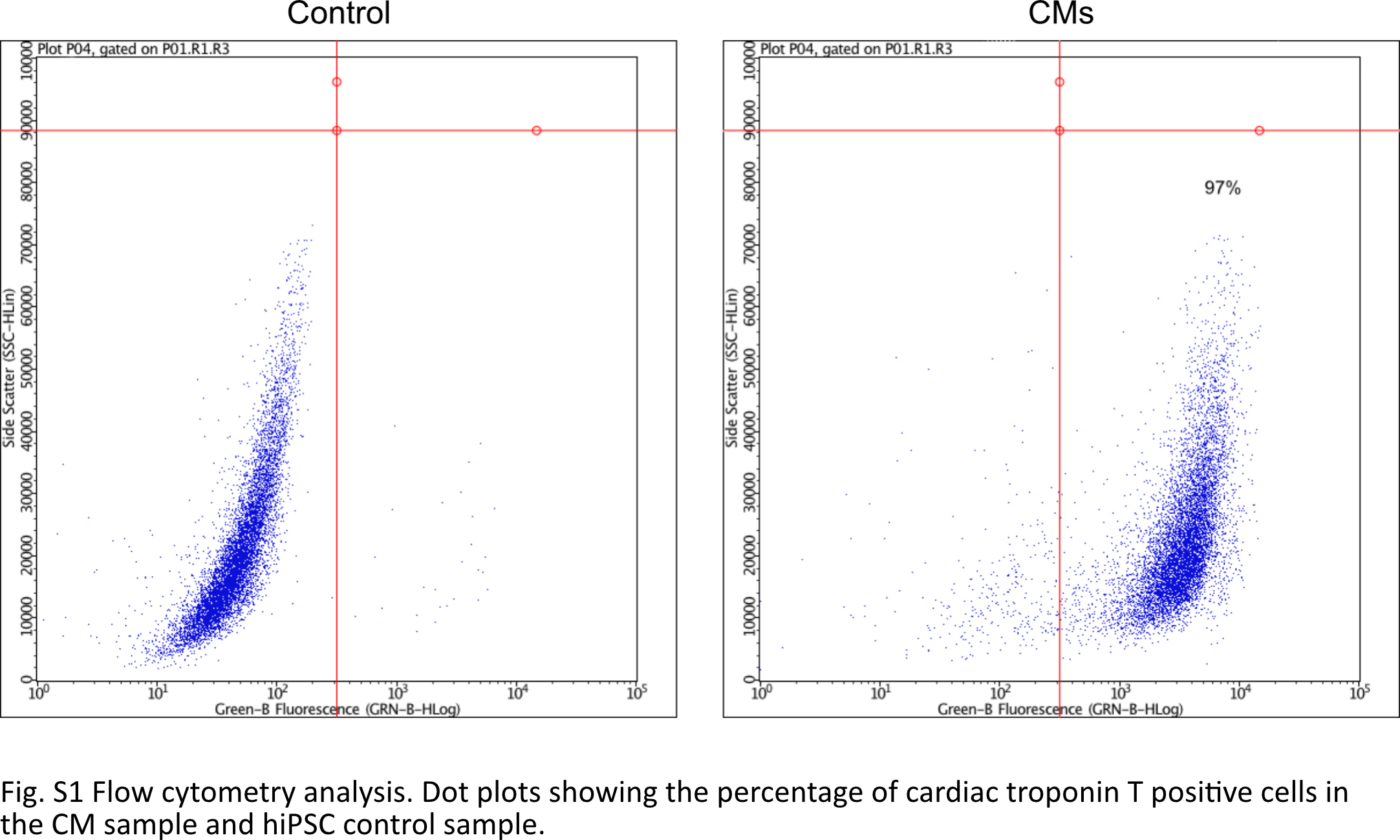

Supplement: Supplementary file 1 [file life-12-00293-s001.zip › life-1577086-supplementary/Figure S2.jpg]

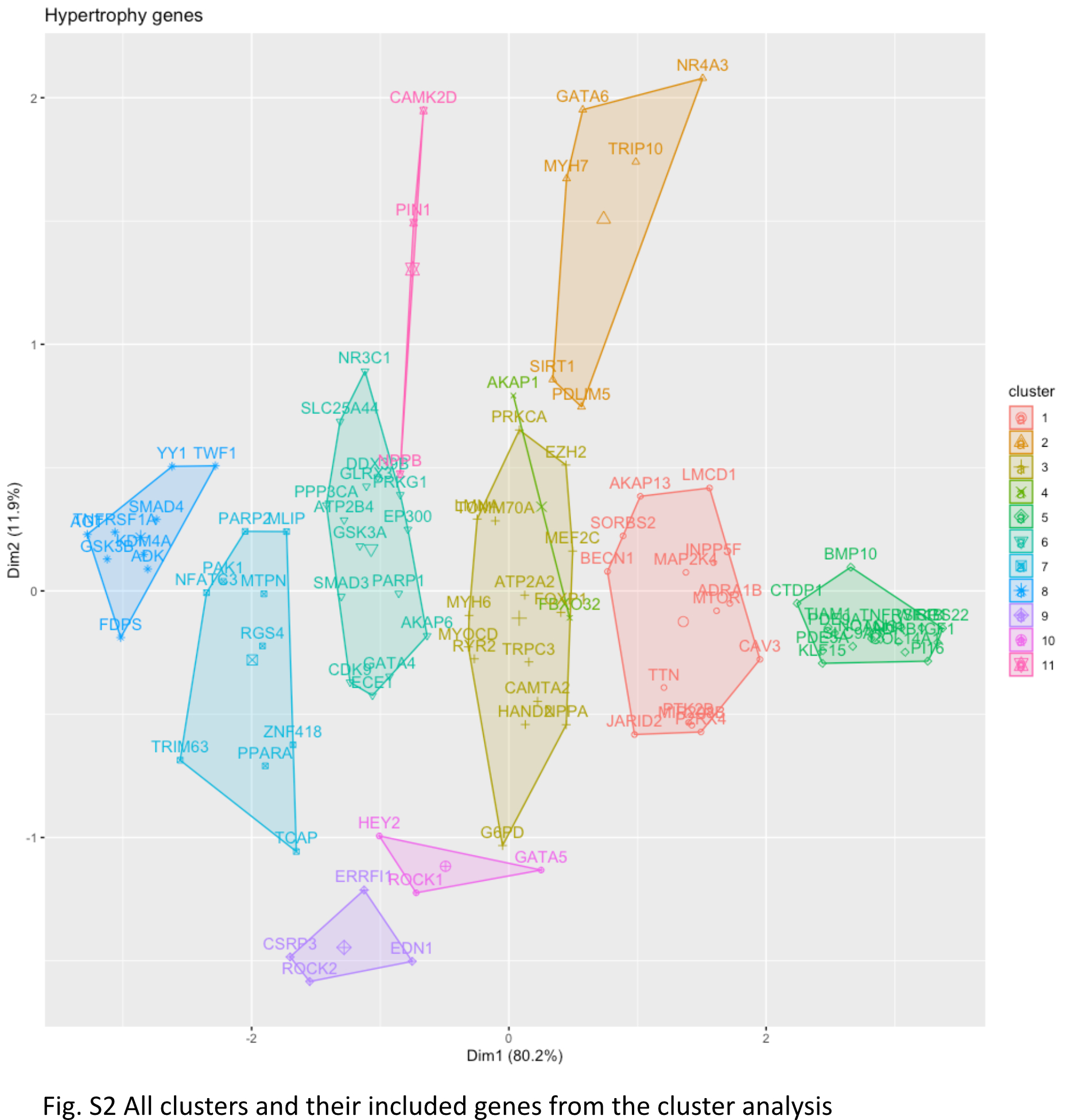

Supplement: Supplementary file 1 [file life-12-00293-s001.zip › life-1577086-supplementary/Figure S3.jpg]
